# Evaluation of the specificity of an intradermal test with recombinant tuberculosis allergen in Bacillus Calmette–Guérin-vaccinated healthy volunteers

**DOI:** 10.3389/fmed.2023.1042461

**Published:** 2023-03-01

**Authors:** Irina A. Vasilyeva, Valentina A. Aksenova, Alexey V. Kazakov, Yulia Y. Kiseleva, Andrey O. Maryandyshev, Elena N. Dolzhenko, Anna V. Abramchenko, Nadejda I. Klevno, Konstantin A. Glebov, Anna E. Panova, Larisa Y. Petrova, Elena G. Sheikis, Inna V. Seregina, Elena I. Nikishova, Natalia P. Doktorova, Anastasia G. Samoilova

**Affiliations:** ^1^FSBI National Medical Research Center for Phthisiopulmonology and Infectious Diseases, Ministry of Health of the Russian Federation, Moscow, Russia; ^2^Department of Phthisiology, Pirogov Russian National Research Medical University, Ministry of Health of the Russian Federation, Moscow, Russia; ^3^Department of Phthisiopulmonology and Thoracic Surgery named after M.I. Perelman, I.M. Sechenov First Moscow State Medical University (Sechenov University), Moscow, Russia; ^4^BHI OR Orlovsky Anti-TB Dispensary, Orel, Russia; ^5^Department of Phthisiopulmonology Northern State Medical University, Arkhangelsk, Russia; ^6^BGI RR Regional Clinical Anti-TB Dispensary, Ryazan, Russia

**Keywords:** tuberculosis, specificity, Diaskintest®, skin test, recombinant tuberculosis allergen

## Abstract

**Introduction:**

The tuberculin skin test has significant limitations for use in individuals vaccinated with BCG. The presence in the genome of *Mycobacterium tuberculosis* of the RDI region, which is absent in the genome of Mycobacterium bovis BCG and most non-tuberculous mycobacteria, made it possible to develop new skin tests, which include a skin test with a recombinant tuberculosis allergen [RTA (Diaskintest®, JSC Generium, Russia)]. Diaskintest has shown high diagnostic performance in clinical trials and in conditions of high prevalence of tuberculosis infection. In 2021, the Russia was excluded from the WHO list of high TB burden countries, which makes relevant an assessment of the specificity of the RTA test under conditions of low epidemiologic risk for tuberculosis to confirm the high specificity of the test.

**Study objective:**

To assess the specificity of Diaskintest in the regions of the Russian Federation with low epidemiologic risk for tuberculosis.

**Methods:**

A multicenter, open-label, prospective study was conducted, which included 150 healthy volunteers aged 18–30 years old, vaccinated with BCG, who were not at risk of tuberculosis, from regions with low epidemiologic risk (Oryol region, Ryazan region, and Arkhangelsk region). During the study, 4 visits were scheduled for each participant: [Visit 0 (screening), Visit 1, Visit 2 (in 72 h) and Visit 3 (in 28 days)]. All participants, who excluded active and latent tuberculosis infection, underwent a test with RTA. To assess the safety of RTA tests, all systemic and local adverse events that occurred during 28 days were recorded. The trial was filed in the NIH clinical trials database ClinicalTrials.gov (NCT05203068).

**Results:**

In individuals with a negative T-SPOT.TB test, the specificity of the RTA test was 97% (95% CI: 92–99%) with a cut-off of >0 mm. The study findings confirm data 2009: 100.00 (95% CI: 94–100). When evaluating the safety of the RTA test during 28 days of follow-up, the participants did not report local and systemic adverse reactions that had a causal relationship with the RTA test.

**Conclusion:**

Diaskintest is highly specific and safe, therefore it is a valuable tool as a screening test for early detection of tuberculosis.

## Introduction

Tuberculosis is the most common infection affecting over 10 million people annually and causing about 1.2 million deaths (1). Tuberculin has been used worldwide for the early detection of tuberculosis infection for a century. The main disadvantage of the tuberculin skin test (TST) is the high number of false positive reactions associated with cross-reactions of tuberculin antigens, which are found in many types of non-tuberculous mycobacteria (NTMs) and Bacillus Calmette–Guérin (BCG) strains. According to a meta-analysis of 14 foreign studies, the cumulative sensitivity of the tuberculin test was 71%, while the specificity was 65.9% (2). The limited specificity of the tuberculin skin test (TST) is especially important due to the wide use of BCG vaccination and a large reservoir of tuberculosis infection, i.e., for most countries with an increased incidence of tuberculosis. Farhat et al. (3), in a review of 12 studies involving 12,728 subjects vaccinated with BCG in infancy, established that BCG was the cause of false-positive tuberculin skin test in 40% of participants and 20% after vaccination 10 years ago or more. In 18 studies involving 1,169,105 subjects, the absolute prevalence of false-positive tuberculin test results due to cross-reactivity with NTMs varied from 0.1 to 2.3% in different regions (3).

The discovery of antigens specific for *Mycobacterium tuberculosis* that are absent in *Mycobacterium bovis* BCG and most mycobacterial species led to the development of *in vitro* tests based on the measurement of Interferon-Gamma Release Assays (IGRA) in response to stimulation by these antigens. The genes encoding these proteins are located in the genomic RD1 region (*region of difference*), which is absent in the genomes of *Mycobacterium bovis* BCG and most non-tuberculous mycobacteria. IGRA tests showed high sensitivity and almost absolute specificity. According to Abubakar et al. (4) comparing the predictive value of positive and negative results of various tests showed that a positive T-SPOT.TB result was a significantly better predictor of progression of active tuberculosis than all other tests (QuantiFERON-TB Gold In-Tube, TST at a cut-off of 15 mm, 10 mm and 5 mm): 4.2, 3.3, 3.5, 2.7, 2.2%, respectively, with the lowest prognostic values found in TST at a cut-off of 5 mm.

Given the high cost-effectiveness of skin tests, there is a need to develop new innovative *in vivo* products. At the same time, it is important that diagnostic tools are tested not only for accuracy, but also for clinical impact and feasibility in resource-limited settings (5).

In 2008, a diagnostic test based on recombinant tuberculosis allergen (RTA), a recombinant CFP10-ESAT6 protein produced by *Escherichia coli* BL21(DE3)/pCFP-ESAT (Diaskintest®, JSC Generium, Russia), was approved in Russia (6). The RTA test is widely used in Russia and the countries of the Commonwealth of Independent States (CIS) for TB screening among children and adolescents as part of the regulatory framework for the early detection of tuberculosis (7–10). More than 60 million tests have been done since the approval of the RTA (11.08.2008, LSR-006435/08^1^).

A review by Krutikov et al. (11) presented a meta-analysis of data on currently available skin tests worldwide. The sensitivity of Diaskintest was 91.18% (95% CI 81.72–95.98) vs. 88.24% (95% CI 78.20–94.01) for the 5 mm TST, and was higher than for other skin tests approved worldwide. In a mixed cohort of individuals with and without tuberculosis, the agreement between the results of the Diaskintest and IGRA tests was 87.16% (95% CI 79.47–92.24), and the agreement with the results of the 5 mm cut-off TST was 55.45% (95% CI 46.08–64.45). The specificity of Diaskintest was not assessed in this meta-analysis since tuberculosis infection was not ruled out in the included populations, and the studies were conducted in high-prevalence settings (11). It should be noted that the specificity of the test is one of the main characteristics of diagnostic tests and refers to the ability of the test to correctly identify subjects without the disease.

In 2021, the Russian Federation (RF) was excluded from the World Health Organization (WHO) list of high TB burden countries (1), which makes relevant an assessment of the specificity of the RTA test in Russian regions with low epidemiologic risk for tuberculosis to confirm the high specificity of the test.

The objective of the study was to evaluate the specificity of the RTA test (Diaskintest) in the regions of the Russian Federation with low epidemiologic risk for tuberculosis.

## Methods

### Study design and participants

This was a prospective, multicenter, open-label, non-comparative study. This study was conducted in the Russian Federation from December 2021 to January 2022 and included 150 healthy volunteers aged 18–30 who were not at risk for developing tuberculosis, from regions with low epidemiologic risk for tuberculosis.

Inclusion criteria: signed informed consent for the study; access for investigators to medical records; age from 18 to 30 years; a history of BCG vaccination (confirmed by medical records and/or presence of a BCG scar); healthy according to the physical examination and medical records at screening; willingness to cooperate and follow the investigator’s recommendations in accordance with the protocol.

Exclusion criteria: a history of tuberculosis or close contact with a patient with active tuberculosis within 5 years prior to inclusion in the study; positive T-SPOT.TB result; use of drugs that affect the immune system within 3 months prior to inclusion in the study; vaccinated against any infections <1.5 months prior to the enrollment date; vaccinated with BCG <6 months prior to the enrollment date; Mantoux test with 2 TU and/or recombinant tuberculosis allergen test was performed within less than 6 months prior to the enrollment date; congenital or acquired immunodeficiency; active immune disease; HIV infection; current condition of the skin that prevents performing and reading of skin tests (injuries, skin diseases); a disease in which blood sampling poses a risk to the participant (hemophilia, other bleeding disorders) or difficult venous access; current participation in another clinical study of the medicinal product or participation in another clinical study within 3 months prior to enrollment in the study; previous participation in clinical studies of ESAT-6 and/or CFP-10 antigens; pregnancy, breastfeeding, planned pregnancy; unwillingness of a woman to use effective methods of contraception during the study; a history of alcohol, drug, benzodiazepine or other substance abuse within 12 months prior to enrollment in the study; consumption of alcohol within 24 h before the visit; a condition or disease that, in the opinion of the investigator, makes the subject not eligible for participation in the study.

Withdrawal criteria: refusal to participate in the study.

During the study, 4 visits were scheduled for each participant: [Visit 0 (screening), Visit 1, Visit 2 (in 72 h) and Visit 3 (in 28 days)]. At the screening stage, an initial examination was carried out, which included obtaining informed consent, assigning an individual number, collecting a history (including allergies), complaints, collecting information on inclusion/exclusion criteria, physical examination, including measurement of height and body weight, measurement of heart rate (HR), respiratory rate (RR), blood pressure (BP), and venous blood sampling for the T-SPOT.TB test.

At Visit 1, in case of a negative T-SPOT.TB test, it was planned to conduct an intradermal RTA test in accordance with the current regulatory documents in the Russian Federation, as well as an assessment of adverse events after administration of RTA.

At Visit 2 (72 h after Visit 1), the response to the intradermal RTA test and development of adverse events after the administration of RTA for the intradermal test were assessed.

Visit 3 was carried out 28 days after Visit 1 and included an assessment of the presence of adverse events that arose after an intradermal RTA test (collection of complaints).

Each participant was under observation throughout the study, including be means of modern telecommunication technologies.

The study was conducted at three sites. The following parameters were used to select the regions: the region status based on the annual ranking of the regions of the Russian Federation by the main epidemic indicators; the incidence rate in children and adolescents (as a true marker of low spread of tuberculosis in the region); opinion of 3 independent leading experts on anti-tuberculosis care in Russia. The cut-off of epidemiological indicators used to select the regions: the incidence of children is not more than 2.0 per 100 thousand children, the incidence of adolescents is not more than 3.5 per 100 thousand of the adolescent population. Three territories were identified: Oryol Oblast, Ryazan Oblast and Arkhangelsk Oblast (Table 1).

**Table 1:** Results of assessing the regions with low epidemiologic risk for tuberculosis.

Region	Rank	Incidence rate in children, per 100,000	Incidence rate in adolescents, per 100,000	Expert opinion			Final decision on inclusion of the territory in the study
				Expert 1	Expert 2	Expert 3	
Belgorod Region	1	1.2	4.6	Yes	Yes	Yes	No
Kostroma Region	2	4.6	5.1	Yes	Yes	Yes	No
Moscow	3	2.3	3.5	No	Yes	Yes	No
Arhangelsk Region	4	0.5	0	Yes	Yes	Yes	Yes
Oryol Region	5	1.8	0	Yes	Yes	Yes	Yes
Nenets Autonomous Region	6	0	0	No	Yes	No	No
Voronezh Region	7	2	16.2	Yes	Yes	Yes	No
Vologda Region	8	0.5	2.7	No	Yes	Yes	No
Moscow Region	9	4.1	6.7	Yes	Yes	Yes	No
Kaluga Region	10	9.3	18.3	Yes	Yes	Yes	No
Ryazan Region	11	1.8	3.4	Yes	Yes	Yes	Yes
Republic of Karelia	12	0	0	No	No	Yes	No
Tambov Region	13	3.5	7.2	Yes	Yes	Yes	No
Vladimir Region	14	7.1	5.1	Yes	Yes	Yes	No
Murmansk Region	15	4.6	12.6	Yes	Yes	Yes	No

Risk groups for the development of tuberculosis are defined by regulatory documents of the Russian Federation (9).

Healthy individuals – individuals without clinical symptoms and in the absence of latent tuberculosis infection. The laboratory T-SPOT.TB test was used to rule out latent tuberculosis infection.

The STARD checklist is available as Supplementary information S1.

### Test methods

#### Recombinant tuberculosis allergen (Diaskintest)

Recombinant tuberculosis allergen (RTA, Diaskintest), GENERIUM JSC, Russia, is a recombinant hybrid CFP10-ESAT6 protein produced by *Escherichia coli* BL21(DE3)/pCFP-ESAT. Diaskintest is performed *in vivo* and is based on a delayed-type hypersensitivity reaction after intradermal administration of specific proteins. The test with recombinant tuberculosis allergen (Diaskintest) was performed in accordance with the instructions for use of the drug: RTA at a dose of 0.1 ml was injected intradermally into the inner surface of the middle third of the right or left forearm. For the test, a 1 ml syringe with a short beveled needle (tuberculin syringe) was used. The result was read by a qualified healthcare professional after 72 h by measuring the diameter of the papule at the injection site. As recommended in the instructions for use and regulatory documents in Russia, the results of the RTA test were interpreted as positive in the presence of a papule of any size (cut-off >0 mm) (9, 12).

#### Т-SPOT.ТВ

Before administration of RTA, an *in vitro* test identifying effector T cells that respond to stimulation with *Mycobacterium tuberculosis* antigen was performed using the ELISPOT method (T-SPOT.TB, Oxford Immunotec, United Kingdom) to rule out tuberculosis infection (including latent one). The T-SPOT.TB test was done in accordance with the approved instructions (13). The results were interpreted as follows: positive – the number of spots in a well with ESAT-6 antigen (panel A) and/or CFP-10 antigen (panel B) is 8 to 100, taking into account the number of spots in the blank control; negative – the number of spots in both panels A and B is ≤4, taking into account the results of blank control; borderline – the number of spots in panel A and/or panel B is 5 to 7; uncertain – more than 11 spots in the blank control or the number of spots in the positive control is <20. A positive result of the T-SPOT.TB test indicated that the sample contained effector T cells reactive (specifically sensitized) to *M. tuberculosis.* Negative result indicated that the sample probably did not contain effector T cells reactive to *M. tuberculosis.* Upon receipt of an indeterminate or positive T-SPOT.TB test result, the participant was not included in the study.

#### Sample size calculation

The sample size for the assessment of the specificity was preliminarily calculated as the relative frequency with the binomial distribution. For N subjects, the variance of the estimated specificity is calculated as Var (û) = u (1 – u)/N, where u is the (unknown) true specificity. For a fixed value of N, Var (û) reaches its maximum at *u* = 0.5. With *N* = 150, this maximum is 0.25/*N* = 0.0017, which corresponds to the standard deviation SD (û) = √(Var (û)) = 0.041. Since the true specificity is expected to be above 0.5, a standard deviation of 0.041 is the worst-case scenario. With a more realistic value of the true specificity of 0.9 (the specificity was more than 90% in the studies assessing the RTA specificity), the corresponding standard deviation is equal to 0.024, which is considered acceptable accuracy of the estimate.

#### Data safety monitoring board

To assess the safety of RTA tests, all systemic and local adverse events that occurred during 28 days were recorded. The study protocol planned to collect data on the following parameters: HR (bpm), SYST BP (mm Hg), DIAST BP (mm Hg), temperature (°C), the presence of general, respiratory complaints, physical examination of the study participants.

### Ethics statement

The study was approved by the local ethics committee of the FSBI National Medical Research Center of Phthisiopulmonology and Infectious Diseases under the Ministry of Health of Russia on November 22, 2021 (Protocol No. 102/1). Before participating in the study, all participants signed a written informed consent. This study was conducted in accordance with the applicable ethical principles described in the Declaration of Helsinki and the requirements of Good Clinical Practice (14). The trial was filed in the NIH clinical trials database ClinicalTrials.gov (NCT05203068). The full study protocol is available at ClinicalTrials.gov.

### Statistical analysis

Data collection, their subsequent correction, systematization of source information and visualization of the obtained results were carried out in Microsoft Office Excel (2016) spreadsheets. MSO (version 2,109 16.0.14430.20154) 32-bit Product number: 00333–59,091-97,459-AA884. Statistical processing of the results was carried out using the Python language (Python 3.8.). Built-in functions from the Scipy module were used for calculations.

Quantitative parameters were evaluated the normality of distribution using the Shapiro–Wilk test. Testing for the normality of distribution showed that the data in the study were not normally distributed. Therefore, further calculations were made using nonparametric statistics methods.

Sets of quantitative parameters were described using the values of the median (Me) and the lower and upper quartiles [Q1; Q3]. The Wilcoxon W test was used to test for differences between the two compared paired samples. The nonparametric Friedman test was used to compare more than two dependent populations. The Mann–Whitney *U*-test was used to compare unrelated samples.

Nominal data were described by absolute and relative (%) values. For qualitative and categorical parameters (sex, etc.), absolute values will be presented in the n/N format, as well as proportions (%), where N is the number of non-missing values, and n is the number of values with the presence of the observed sign. Comparison of nominal data was carried out using Pearson’s chi-squared test. In cases where the number of expected observations in any of the cells of the four-field table was less than 10, the Fisher’s exact test was used to assess the level of significance of differences. Comparison of nominal data in dependent groups was carried out using the McNemar’s chi-squared test.

Specificity measures the proportion of correctly identified negative results (e.g., the percentage of healthy people who are correctly identified as having no disease). Specificity, or TNR (True Negative Rate), shows the accuracy of prediction/determination of 0 and is calculated using the following formula TNR = TN /(TN + FP), where.

True negative (TN) result is the number of correctly classified negative samples. True negative results.

False positive (FP) result is the number of falsely classified positive examples. False positive results.

Specificity was reported with indication of percentage and 95% CI calculated using the Wilson method [LB; UB]. Comparison of specificity in different years was carried out using the Pearson’s chi-squared test or the Fisher’s exact test.

Differences were considered statistically significant at *p* < 0.05. The obtained value of *p* > 0.05 indicated the absence of statistically significant differences. The value of *p* < 0.05 indicates their presence.

## Results

**Figure 1:**
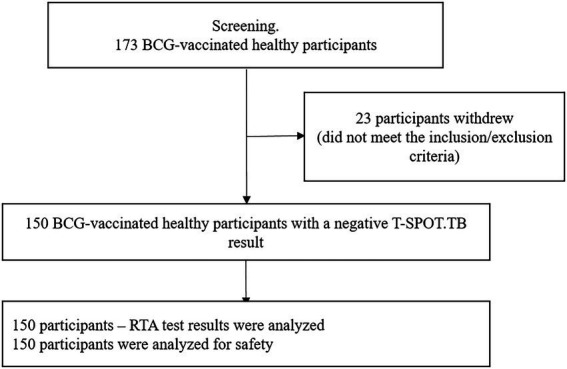
Block diagram of the study of the specificity of RTA test.

The study included 150 healthy, BCG-vaccinated subjects. RTA test results and safety analyses were performed for all participants (Figure. 1). The time between visit 0 and visit 1 (Time interval between blood sampling for T-SPOT.TB test and skin test with RTA) was no more than 24 h.

Baseline demographic and clinical characteristics of participants: women predominated – 122/150 (81.3%), the mean age was 21.5 years (95% CI 20.0–22.0), the body mass index was within normal limits – 21.6 (95% CI 19.9–24.1). The majority of participants [145 (96.7%)] were vaccinated against COVID-19 more than 1.5 months ago.

2/150 (1.3%) of the study participants had allergic diseases in remission, without exposure to an allergen for a long time, 8/150 (5.3%) had chronic diseases in stable remission (myopia in 5/8, hiatal hernia in 1/8, cardiovascular disorder in 2/8). In 2/150 (1.3%), an objective examination revealed arrhythmia and systolic murmur. All these conditions did not affect the immune response and were the criteria for exclusion of participants from the study (Table 2).

**Table 2:** Characteristics of subjects included in the study.

Parameter	Subjects, *n* = 150
Sex (%)	
Women	122 (81.3)
Men	28 (18.7)
Weight, kg	60.0
Height, m	1.7
BMI	21.6
Age (years)	22.0
BCG vaccination (%)	
No	0 (0)
Yes	150 (100.0)
COVID19 vaccination (%)	
No	5 (3.3)
Yes	145 (96.7)
History of allergies (%)	
No	148 (98.7)
Yes	2 (1.3)
Chronic diseases (%)	
No	142 (94.7)
Yes	8 (5.3)

BMI, body mass index.

When assessing changes in the state of the study participants over time, statistically significant differences from visit 0 to visits 1 and 2 were determined only in heart rate and respiratory rate, and the range of values remained within normal, the differences were not clinically significant. HR and RR became lower at visit 2, which can be interpreted as a decrease in anxiety among subjects by visit 2. Changes in parameters over time are provided in Tables 3, 4.

**Table 3:** Dynamics of parameters over time.

Variables	Subjects, *n*	Me	95% Cl		*p* (Friedman’s test, pairwise comparison – Wilcoxon’s *W*-test)
			Lower	Upper	
HR (bpm)					*p* = 0.01*, *p*1–2:=0.319, *p*1–3:<0.001*, *p*2–3:=0.001*
Visit 0	150	78.0	72.0	87.0	
Visit 1	150	78.0	71.0	85.0	
Visit 2	150	74.5	70.0	81.0	
Systolic blood pressure (mmHg)					*p* = 0.108
Visit 0	150	116.5	110.0	127.75	
Visit 1	150	116.0	110.0	126.75	
Visit 2	150	115.0	110.0	120.0	
Diastolic blood pressure (mmHg)					*p* = 0.067
Visit 0	150	75.0	70.0	80.0	
Visit 1	150	75.0	70.0	80.0	
Visit 2	150	75.0	70.0	80.0	
*t*° (С)					*p* = 0.052
Visit 0	150	36.4	36.1	36.6	
Visit 1	150	36.4	36.12	36.6	
Visit 2	149	36.4	36.2	36.5	
Respiratory rate (per minute)					
Visit 0	149	17.0	16.0	18.0	*p* < 0.001*, *p*1–2 = 0.73, *p*1–3 < 0.001*, *p*2–3 < 0.001*
Visit 1	150	17.0	16.0	18.0	
Visit 2	148	16.0	16.0	17.0	

HR, heart rate; bpm, beats per minute. *Statistically significant comparisons (*p* < 0.005).

**Table 4:** Dynamics of parameters over time based on the results of examination of patients.

Variables	Subjects, *n*	Subjects with presence of changes, *n* (%)	*p* (Friedman’s test, pairwise comparison – Wilcoxon’s *W*-test)
General complaints			
Visit 0	150	0 (0.0)	*p* = 0.367,
Visit 1	150	0 (0.0)	*p*1–2 = 1.000,
Visit 2	150	1 (0.7)	*p*1–3 = 0.318,
			*p*2–3 = 0.318
General condition (satisfactory)			
Visit 0	150	150 (100.0)	*p* = 1.0,
Visit 1	150	150 (100.0)	*p*1–2 = 1,
Visit 2	150	150 (100.0)	*p*1–3 = 1,
			*p*2–3 = 1
Respiratory system			
Visit 0	150	0 (0.0)	*p* = 1.0,
Visit 1	150	0 (0.0)	*p*1–2 = 1,
Visit 2	150	0 (0.0)	*p*1–3 = 1,
			*p*2–3 = 1
Сardiovascular system^1^			
Visit 0	150	6 (4.0)	*p* = 0.941,
Visit 1	150	6 (4.0)	*p*1–2 = 1.0,
Visit 2	150	5 (3.3)	*p*1–3 = 0.318,
			*p*2–3 = 0.318
Gastrointestinal system^2^			
Visit 0	150	6 (4.0)	*p* = 0.772,
Visit 1	150	6 (4.0)	*p*1–2 = 1.0,
Visit 2	150	4 (2.7)	*p*1–3 = 0.158,
			*p*2–3 = 0.158
Urinary system (%)			
Visit 0	150	0 (0.0)	*p* = 1.0,
Visit 1	150	0 (0.0)	*p*1–2 = 1,
Visit 2	150	0 (0.0)	*p*1–3 = 1,
			*p*2–3 = 1
Gynecological history (%)			
Visit 0	122	0 (0.0)	*p* = 1.0,
Visit 1	122	0 (0.0)	*p*1–2 = 1,
Visit 2	122	0 (0.0)	*p*1–3 = 1,
			*p*2–3 = 1
Neurological status^3^			
Visit 0	150	1 (0.7)	*p* = 1.0,
Visit 1	150	1 (0.7)	*p*1–2 = 1,
Visit 2	150	1 (0.7)	*p*1–3 = 1,
			*p*2–3 = 1

^1^Disorders of cardiovascular system: dextraposition of the heart (1), functional systolic murmur (5/4). ^2^Disorders of gastrointestinal system: defecation disorder. ^3^Disorders of neurological status: sleep disturbance.

When evaluating the safety of the RTA test, one of the 150 participants had a general adverse event: pyrexia up to 37.8, nausea and muscle aches developed in the evening (at 19:40) on the day of the RTA test. An express PCR test for COVID-19 was performed (negative), no additional treatment was prescribed. The subject got better the next day. This condition was regarded as acute respiratory viral infection, a causal relationship with RTA was not established, the result of the RTA test at visit 2 was assessed as negative (Table 5).

**Table 5:** Presence of adverse events.

Adverse events	Subjects, *n* (%)	*р* (Fisher’s exact test)
Visit 1		*p* = 1.000
1 – absent	150 (100.0)	
2 – mild	0 (0.0)	
3 – moderate	0 (0.0)	
4 – severe	0 (0.0)	
Visit 2		
1 – absent	149 (99.3)	
2 – mild	1 (0.7)	
3 – moderate	0 (0.0)	
4 – severe	0 (0.0)	
Visit 3		
1 – absent	150 (100.0)	
2 – mild	0 (0.0)	
3 – moderate	0 (0.0)	
4 – severe	0 (0.0)	

At Visit 2, the RTA test result was assessed as positive in 5/150 (3.3%) of the subjects. The median papule size was 14.5 (95% CI 11.0–18.8) mm.

When calculating the specificity, the obtained data were used: 145 true negative (TN) results of the RTA test, 5 false positive (FP) test results. Specificity = 145/(145 + 5). Thus, the specificity of the RTA test is 0.97. The confidence interval was calculated using the Wilson method and amounted to (95% CI 0.92–0.99) (Table 6).

**Table 6:** Confusion matrix.

RTA test	T-SPOT.TB test result		Total subjects
	Available	Not available	
Available	0	5	5
Not available	0	145	145
Total	0	150	150

The results of the specificity of RTA test were compared with the 2009 data (15).

**Figure 2:**
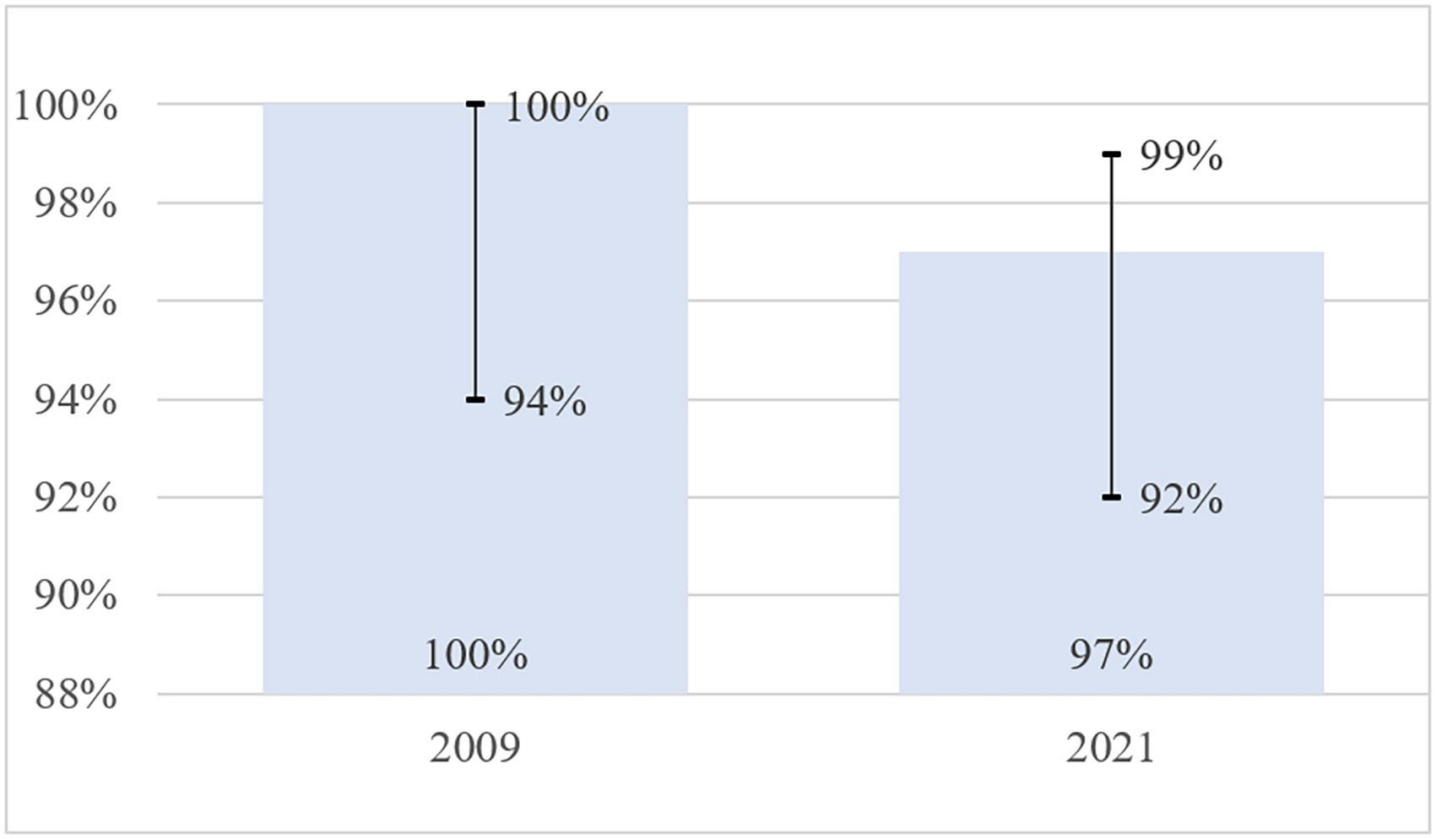
Comparative results of RTA test specificity in 2009 and 2021.

The current study with a large number of participants (*n* = 150) showed that specificity did not change statistically significantly compared to data published in 2009 (*n* = 55) (*p* = 0.898). In 2009, the specificity was 1.00 (95% CI 0.94–1.00), it was 0.97 (95% CI 0.92–0.99) in 2021 (Table 7 and Figure 2).

**Table 7:** Comparative results of RTA test specificity in 2009 and 2021.

Parameter	2009	2021	*p* (Chi-squared test)
Specificity (TNR)	1.00 [95% Cl 0.94; 1.00]	0.97 [95% Cl 0.92; 0.99]	*p* = 0.898
Specificity (TNR), %	100% [95% Cl94%; 100%]	97% [95% Cl 92%; 99%]	*p* = 0.898

## Discussion

The Russian RTA skin test is an analogue of IGRA tests. Clinical studies established high diagnostic parameters of the test: sensitivity 95% CI: 98–100%, specificity 95% CI: 90–100% (16). The sensitivity of the test was 96.5% (95% CI 94.5–97.8) in a continuous study of children and adolescents with tuberculosis diagnosed in Moscow in 2012–2014 (17–19). A study by Bogorodskaya (20) evaluated the results of a RTA test as part of routine clinical practice in individuals at low risk of tuberculosis infection (close contacts of pregnant women) in a region of the Russian Federation with a low incidence of tuberculosis (Moscow). In 2015–2016, a total of 7,249 tests were performed in individuals from this population, which is the most representative of the resident population of Moscow. Positive or equivocal results of the RTA test in individuals who were close contact of pregnant women were found in 0.3% (95% CI 0.2–0.6%) in 2015 and in 1.0% (95% CI 0.7–1.4%) of tested individuals in 2016 (*p* < 0.05). No tuberculosis patients were identified in this group. Thus, the test specificity was 99.0–99.7% (20). The high effectiveness of the test in clinical practice has been confirmed by many authors (21–23). Studies have demonstrated that the use of the RTA test for TB screening has significantly increased the detection rate of tuberculosis in primary health care facilities (in schoolchildren 8–17 years of age, it is 37-fold higher than that with the use of the Mantoux test) (24).

In our RTA specificity study, out of 150 participants with a negative T-SPOT test, a TB positive RTA test was reported in 3.3% of the subjects. A negative result of the T-SPOT.TB test, given the high diagnostic performance of the T-SPOT.TB test, was used as an inclusion criterion to exclude the presence of latent TB infection in participants (4, 13, 25). Therefore, our data suggest the specificity of the RTA test was 97% (95% CI: 92–99%) with a cut-off of >0 mm (an induration of any size at the injection site). The study findings confirm previously published 2009 data on the specificity of RTA test 100.00 (95% CI 94–100) (15).

It should be noted that before inclusion in the study, some participants had conditions and diseases in stable remission, which were not exclusion criteria for participants. During follow-up monitoring of the participants’ condition, it was noted that these conditions and diseases did not affect the RTA test results.

Based on the study results, we can note a fairly high safety of the RTA test, since none of the participants had local and systemic adverse reactions related to the RTA test during the 28 days of follow-up.

### Limitations of the study

In our RTA specificity study, we used the T-SPOT test, given its high diagnostic performance of the T-SPOT.TB test, as an inclusion criterion to rule out the presence of latent TB infection in participants (4, 13, 25). However, there is no gold standard for diagnosing latent TB infection, and we cannot exclude that some positive reactions to the RTA test may be true positive, despite the fact that the study protocol is designed to minimize the risk of latent TB infection in participants (all subjects are not included in the study group). High-risk TB, had not been in contact with a TB patient in the 5 years prior to enrollment in the study, were healthy according to the physical examination and medical records.

## Conclusion

Thus, our data confirm the previously published results of clinical studies on the diagnostic characteristics of RTA test and are an additional argument for the formation of an evidence base consisting of many years of experience in the use of the RTA test in clinical practice in Russia. Combining the high specificity of laboratory tests and the ease of performing and interpreting the result by a single cut-off (cut-off >0 mm (any papule size is considered positive)), the RTA test is a valuable tool for the early detection of tuberculosis.

## Data availability statement

The raw data supporting the conclusions of this article will be made available by the authors, without undue reservation.

## Ethics statement

The studies involving human participants were reviewed and approved by the local ethics committee of the National Medical Research Center of Phthisiopulmonology and Infectious Diseases under the Ministry of Health of Russia on November 22, 2021 (Protocol No. 102/1). The patients/participants provided their written informed consent to participate in this study.

## Author contributions

All authors listed have made a substantial, direct, and intellectual contribution to the work and approved it for publication.

## Conflict of interest

The authors declare that the research was conducted in the absence of any commercial or financial relationships that could be construed as a potential conflict of interest.

## Publisher’s note

All claims expressed in this article are solely those of the authors and do not necessarily represent those of their affiliated organizations, or those of the publisher, the editors and the reviewers. Any product that may be evaluated in this article, or claim that may be made by its manufacturer, is not guaranteed or endorsed by the publisher.

## Footnotes

^1^https://grls.rosminzdrav.ru/Grls_View_v2.aspx?routingGuid=c92439d2-f007-44cb-af2d-d93083d72fe4&t=
